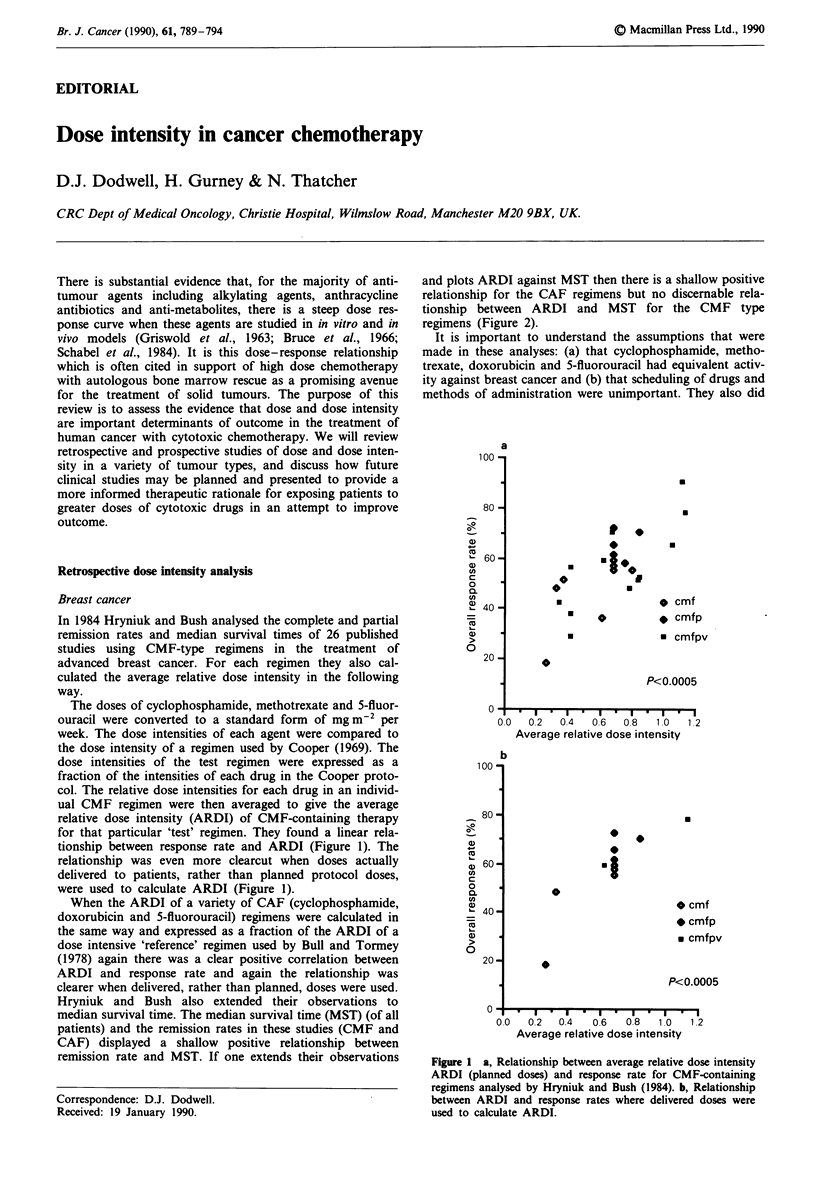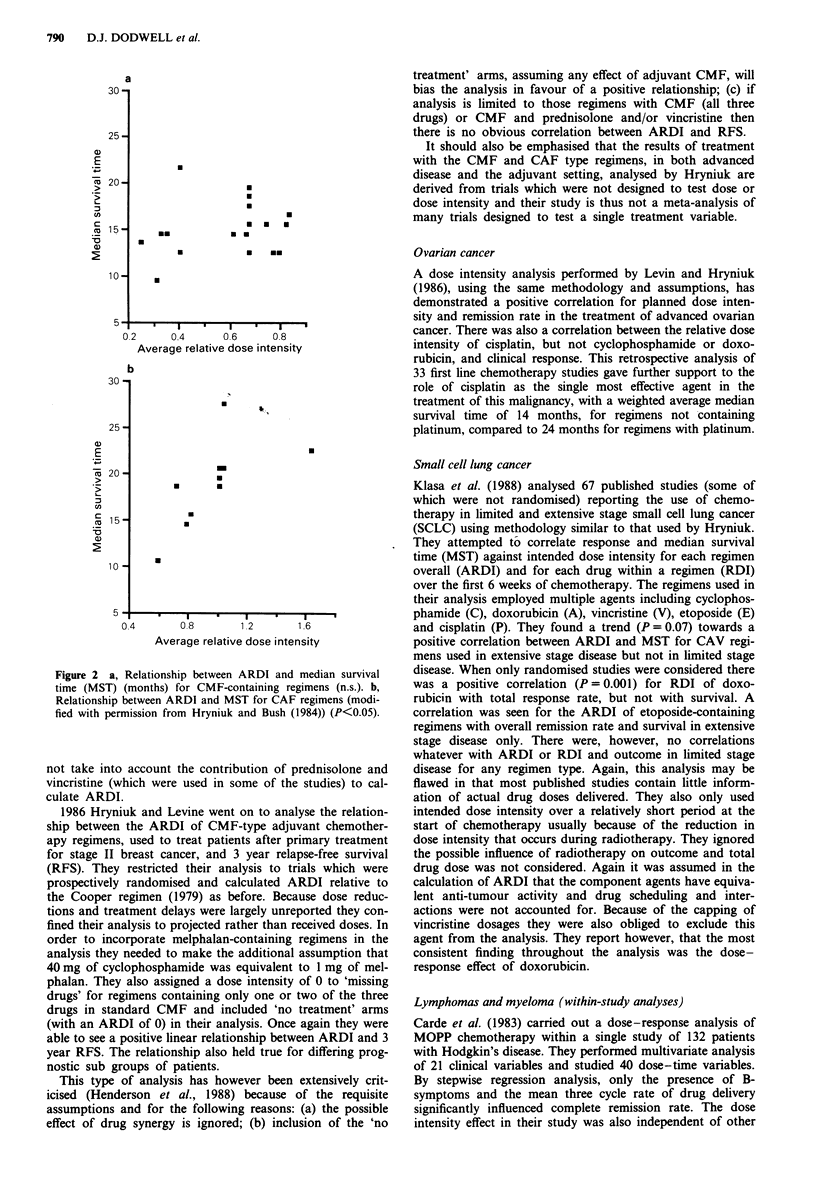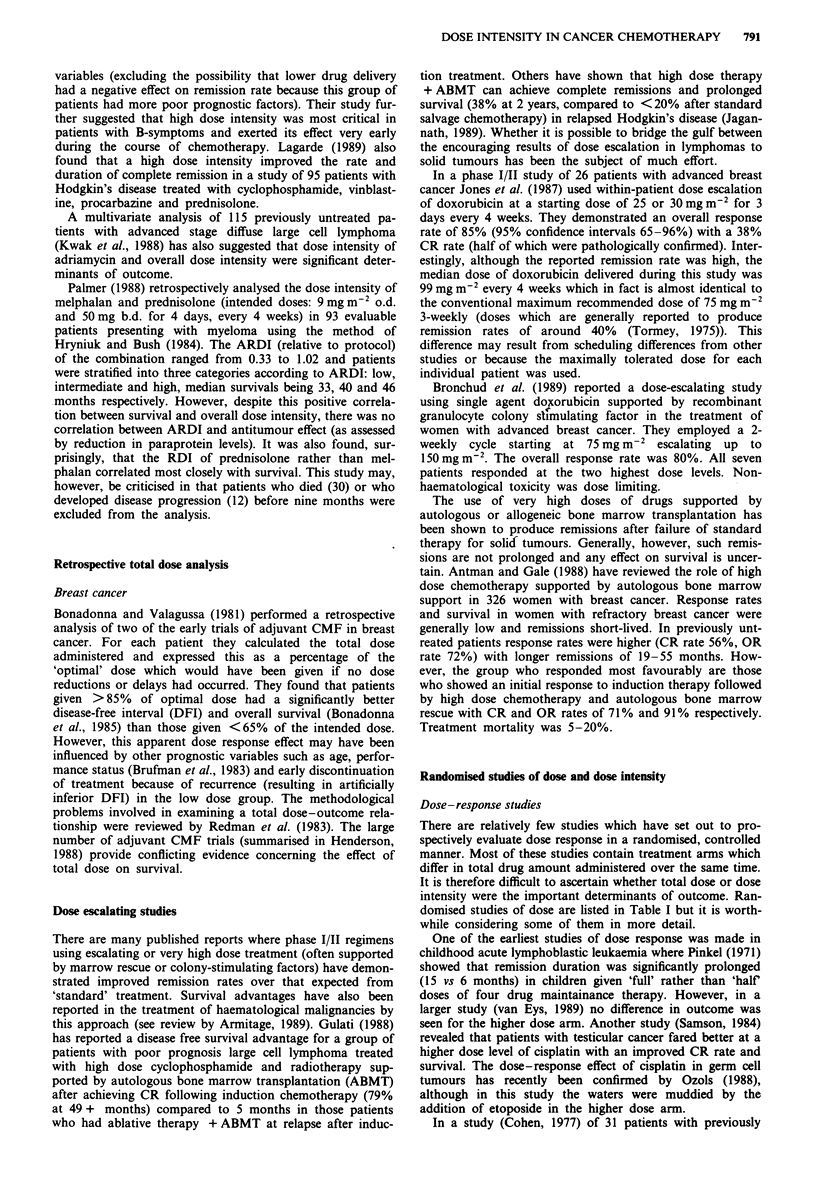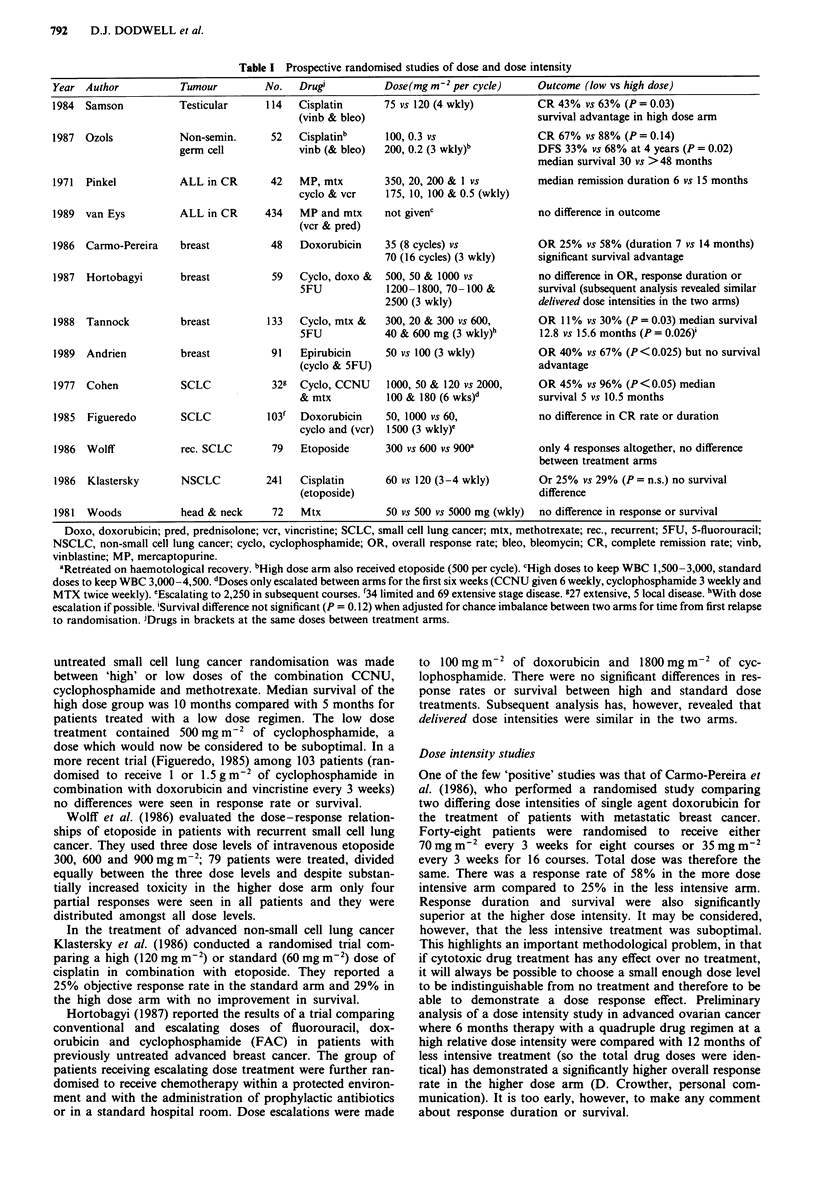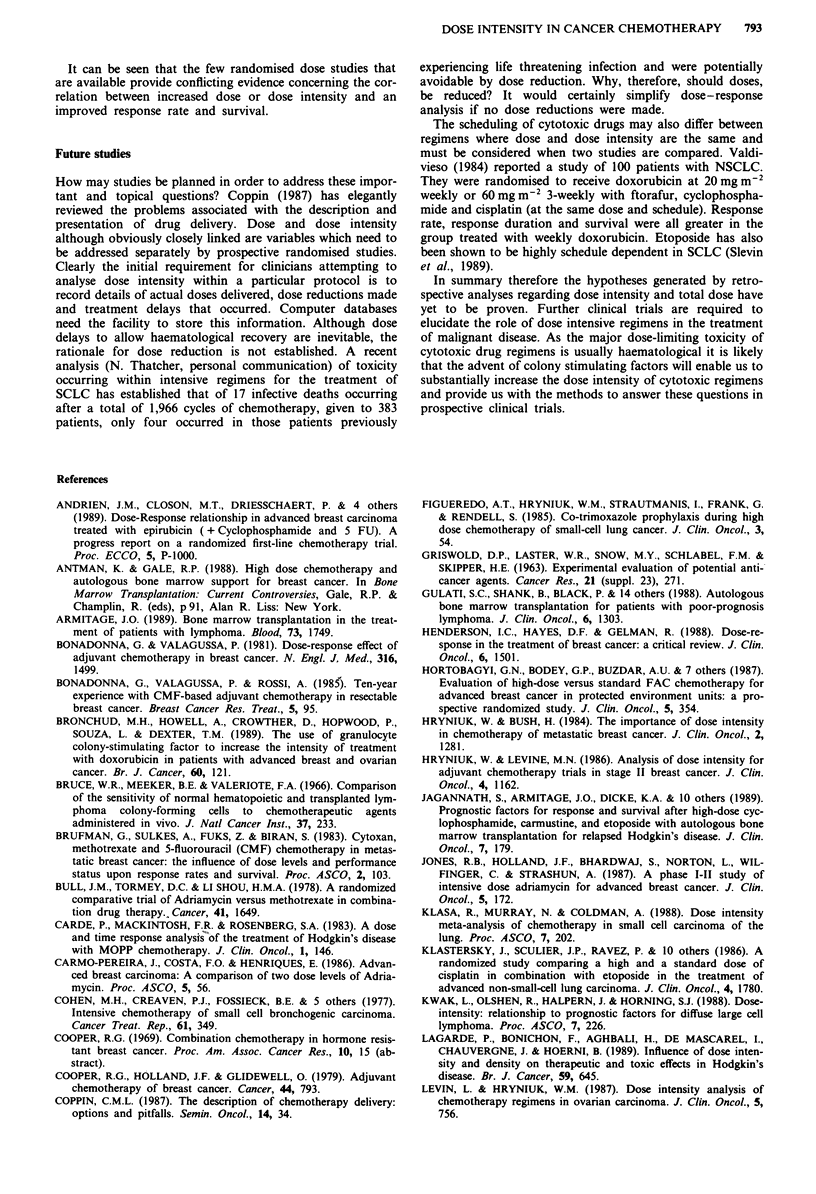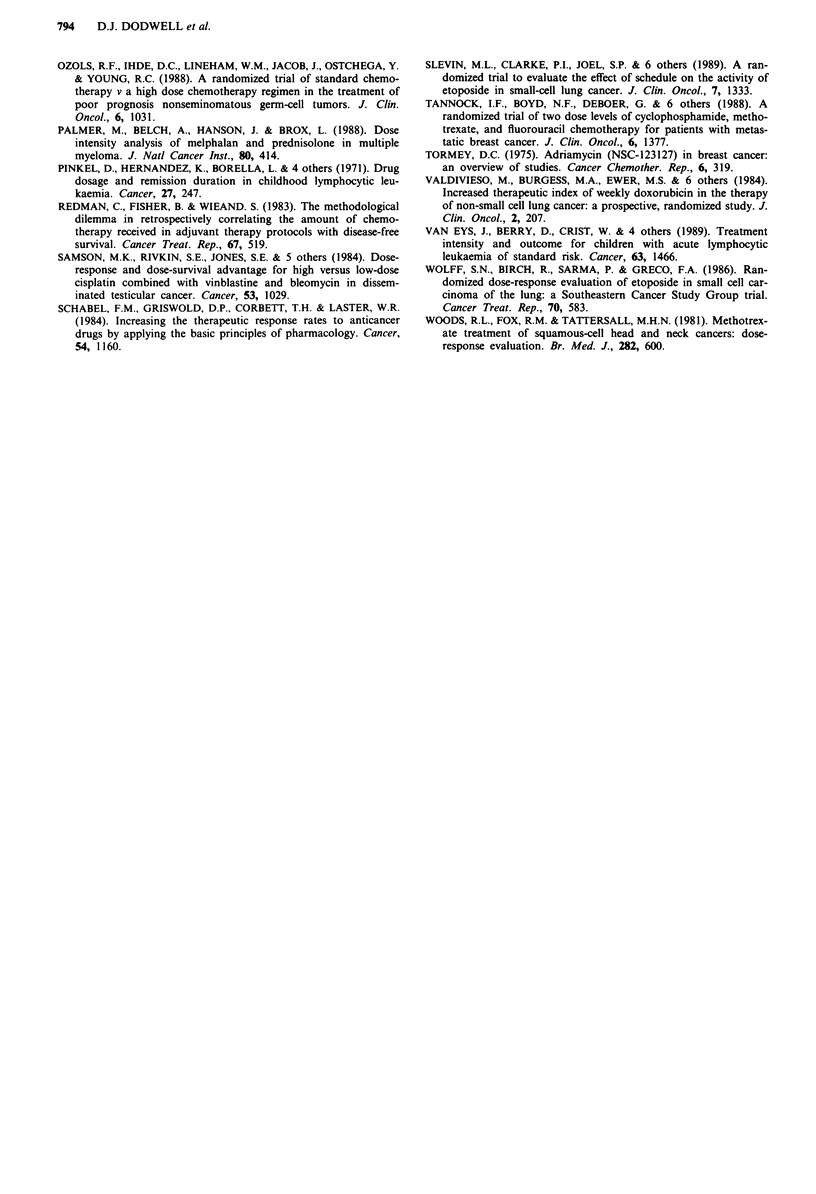# Dose intensity in cancer chemotherapy.

**DOI:** 10.1038/bjc.1990.178

**Published:** 1990-06

**Authors:** D. J. Dodwell, H. Gurney, N. Thatcher

**Affiliations:** CRC Dept of Medical Oncology, Christie Hospital, Manchester, UK.


Br. J. Cancer (1990), 61, 789–794
© Macmillan Press Ltd., 1990

**EDITORIAL**

### Dose intensity in cancer chemotherapy

D.J. Dodwell, H. Gurney & N. Thatcher

*CRC Dept of Medical Oncology, Christie Hospital, Wilmslow Road, Manchester M20 9BX, UK.*

There is substantial evidence that, for the majority of anti-tumour agents including alkylating agents, anthracycline antibiotics and anti-metabolites, there is a steep dose response curve when these agents are studied in *in vitro* and *in vivo* models (Griswold *et al.*, 1963; Bruce *et al.*, 1966; Schabel *et al.*, 1984). It is this dose–response relationship which is often cited in support of high dose chemotherapy with autologous bone marrow rescue as a promising avenue for the treatment of solid tumours. The purpose of this review is to assess the evidence that dose and dose intensity are important determinants of outcome in the treatment of human cancer with cytotoxic chemotherapy. We will review retrospective and prospective studies of dose and dose intensity in a variety of tumour types, and discuss how future clinical studies may be planned and presented to provide a more informed therapeutic rationale for exposing patients to greater doses of cytotoxic drugs in an attempt to improve outcome.

### Retrospective dose intensity analysis

### *Breast cancer*

In 1984 Hryniuk and Bush analysed the complete and partial remission rates and median survival times of 26 published studies using CMF-type regimens in the treatment of advanced breast cancer. For each regimen they also calculated the average relative dose intensity in the following way.

The doses of cyclophosphamide, methotrexate and 5-fluorouracil were converted to a standard form of mg $m^{-2}$ per week. The dose intensities of each agent were compared to the dose intensity of a regimen used by Cooper (1969). The dose intensities of the test regimen were expressed as a fraction of the intensities of each drug in the Cooper protocol. The relative dose intensities for each drug in an individual CMF regimen were then averaged to give the average relative dose intensity (ARDI) of CMF-containing therapy for that particular 'test' regimen. They found a linear relationship between response rate and ARDI (Figure 1). The relationship was even more clearcut when doses actually delivered to patients, rather than planned protocol doses, were used to calculate ARDI (Figure 1).

When the ARDI of a variety of CAF (cyclophosphamide, doxorubicin and 5-fluorouracil) regimens were calculated in the same way and expressed as a fraction of the ARDI of a dose intensive 'reference' regimen used by Bull and Tormey (1978) again there was a clear positive correlation between ARDI and response rate and again the relationship was clearer when delivered, rather than planned, doses were used. Hryniuk and Bush also extended their observations to median survival time. The median survival time (MST) (of all patients) and the remission rates in these studies (CMF and CAF) displayed a shallow positive relationship between remission rate and MST. If one extends their observations and plots ARDI against MST then there is a shallow positive relationship for the CAF regimens but no discernable relationship between ARDI and MST for the CMF type regimens (Figure 2).

It is important to understand the assumptions that were made in these analyses: (a) that cyclophosphamide, methotrexate, doxorubicin and 5-fluorouracil had equivalent activity against breast cancer and (b) that scheduling of drugs and methods of administration were unimportant. They also did

**Figure 1** **a**, Relationship between average relative dose intensity ARDI (planned doses) and response rate for CMF-containing regimens analysed by Hryniuk and Bush (1984). **b**, Relationship between ARDI and response rates where delivered doses were used to calculate ARDI.

Correspondence: D.J. Dodwell.
Received: 19 January 1990.

**Figure 2** **a**, Relationship between ARDI and median survival time (MST) (months) for CMF-containing regimens (n.s.). **b**, Relationship between ARDI and MST for CAF regimens (modified with permission from Hryniuk and Bush (1984)) ($P<0.05$).

not take into account the contribution of prednisolone and vincristine (which were used in some of the studies) to calculate ARDI.

1986 Hryniuk and Levine went on to analyse the relationship between the ARDI of CMF-type adjuvant chemotherapy regimens, used to treat patients after primary treatment for stage II breast cancer, and 3 year relapse-free survival (RFS). They restricted their analysis to trials which were prospectively randomised and calculated ARDI relative to the Cooper regimen (1979) as before. Because dose reductions and treatment delays were largely unreported they confined their analysis to projected rather than received doses. In order to incorporate melphalan-containing regimens in the analysis they needed to make the additional assumption that 40 mg of cyclophosphamide was equivalent to 1 mg of melphalan. They also assigned a dose intensity of 0 to 'missing drugs' for regimens containing only one or two of the three drugs in standard CMF and included 'no treatment' arms (with an ARDI of 0) in their analysis. Once again they were able to see a positive linear relationship between ARDI and 3 year RFS. The relationship also held true for differing prognostic sub groups of patients.

This type of analysis has however been extensively criticised (Henderson *et al.*, 1988) because of the requisite assumptions and for the following reasons: (a) the possible effect of drug synergy is ignored; (b) inclusion of the 'no treatment' arms, assuming any effect of adjuvant CMF, will bias the analysis in favour of a positive relationship; (c) if analysis is limited to those regimens with CMF (all three drugs) or CMF and prednisolone and/or vincristine then there is no obvious correlation between ARDI and RFS.

It should also be emphasised that the results of treatment with the CMF and CAF type regimens, in both advanced disease and the adjuvant setting, analysed by Hryniuk are derived from trials which were not designed to test dose or dose intensity and their study is thus not a meta-analysis of many trials designed to test a single treatment variable.

### *Ovarian cancer*

A dose intensity analysis performed by Levin and Hryniuk (1986), using the same methodology and assumptions, has demonstrated a positive correlation for planned dose intensity and remission rate in the treatment of advanced ovarian cancer. There was also a correlation between the relative dose intensity of cisplatin, but not cyclophosphamide or doxorubicin, and clinical response. This retrospective analysis of 33 first line chemotherapy studies gave further support to the role of cisplatin as the single most effective agent in the treatment of this malignancy, with a weighted average median survival time of 14 months, for regimens not containing platinum, compared to 24 months for regimens with platinum.

### *Small cell lung cancer*

Klasa *et al.* (1988) analysed 67 published studies (some of which were not randomised) reporting the use of chemotherapy in limited and extensive stage small cell lung cancer (SCLC) using methodology similar to that used by Hryniuk. They attempted to correlate response and median survival time (MST) against intended dose intensity for each regimen overall (ARDI) and for each drug within a regimen (RDI) over the first 6 weeks of chemotherapy. The regimens used in their analysis employed multiple agents including cyclophosphamide (C), doxorubicin (A), vincristine (V), etoposide (E) and cisplatin (P). They found a trend ($P = 0.07$) towards a positive correlation between ARDI and MST for CAV regimens used in extensive stage disease but not in limited stage disease. When only randomised studies were considered there was a positive correlation ($P = 0.001$) for RDI of doxorubicin with total response rate, but not with survival. A correlation was seen for the ARDI of etoposide-containing regimens with overall remission rate and survival in extensive stage disease only. There were, however, no correlations whatever with ARDI or RDI and outcome in limited stage disease for any regimen type. Again, this analysis may be flawed in that most published studies contain little information of actual drug doses delivered. They also only used intended dose intensity over a relatively short period at the start of chemotherapy usually because of the reduction in dose intensity that occurs during radiotherapy. They ignored the possible influence of radiotherapy on outcome and total drug dose was not considered. Again it was assumed in the calculation of ARDI that the component agents have equivalent anti-tumour activity and drug scheduling and interactions were not accounted for. Because of the capping of vincristine dosages they were also obliged to exclude this agent from the analysis. They report however, that the most consistent finding throughout the analysis was the dose-response effect of doxorubicin.

### *Lymphomas and myeloma (within-study analyses)*

Carde *et al.* (1983) carried out a dose-response analysis of MOPP chemotherapy within a single study of 132 patients with Hodgkin's disease. They performed multivariate analysis of 21 clinical variables and studied 40 dose-time variables. By stepwise regression analysis, only the presence of B-symptoms and the mean three cycle rate of drug delivery significantly influenced complete remission rate. The dose intensity effect in their study was also independent of other variables (excluding the possibility that lower drug delivery had a negative effect on remission rate because this group of patients had more poor prognostic factors). Their study further suggested that high dose intensity was most critical in patients with B-symptoms and exerted its effect very early during the course of chemotherapy. Lagarde (1989) also found that a high dose intensity improved the rate and duration of complete remission in a study of 95 patients with Hodgkin's disease treated with cyclophosphamide, vinblastine, procarbazine and prednisolone.

A multivariate analysis of 115 previously untreated patients with advanced stage diffuse large cell lymphoma (Kwak *et al.*, 1988) has also suggested that dose intensity of adriamycin and overall dose intensity were significant determinants of outcome.

Palmer (1988) retrospectively analysed the dose intensity of melphalan and prednisolone (intended doses: 9 mg $m^{-2}$ o.d. and 50 mg b.d. for 4 days, every 4 weeks) in 93 evaluable patients presenting with myeloma using the method of Hryniuk and Bush (1984). The ARDI (relative to protocol) of the combination ranged from 0.33 to 1.02 and patients were stratified into three categories according to ARDI: low, intermediate and high, median survivals being 33, 40 and 46 months respectively. However, despite this positive correlation between survival and overall dose intensity, there was no correlation between ARDI and antitumour effect (as assessed by reduction in paraprotein levels). It was also found, surprisingly, that the RDI of prednisolone rather than melphalan correlated most closely with survival. This study may, however, be criticised in that patients who died (30) or who developed disease progression (12) before nine months were excluded from the analysis.

### Retrospective total dose analysis

*Breast cancer*

Bonadonna and Valagussa (1981) performed a retrospective analysis of two of the early trials of adjuvant CMF in breast cancer. For each patient they calculated the total dose administered and expressed this as a percentage of the 'optimal' dose which would have been given if no dose reductions or delays had occurred. They found that patients given >85% of optimal dose had a significantly better disease-free interval (DFI) and overall survival (Bonadonna *et al.*, 1985) than those given <65% of the intended dose. However, this apparent dose response effect may have been influenced by other prognostic variables such as age, performance status (Brufman *et al.*, 1983) and early discontinuation of treatment because of recurrence (resulting in artificially inferior DFI) in the low dose group. The methodological problems involved in examining a total dose–outcome relationship were reviewed by Redman *et al.* (1983). The large number of adjuvant CMF trials (summarised in Henderson, 1988) provide conflicting evidence concerning the effect of total dose on survival.

### Dose escalating studies

There are many published reports where phase I/II regimens using escalating or very high dose treatment (often supported by marrow rescue or colony-stimulating factors) have demonstrated improved remission rates over that expected from 'standard' treatment. Survival advantages have also been reported in the treatment of haematological malignancies by this approach (see review by Armitage, 1989). Gulati (1988) has reported a disease free survival advantage for a group of patients with poor prognosis large cell lymphoma treated with high dose cyclophosphamide and radiotherapy supported by autologous bone marrow transplantation (ABMT) after achieving CR following induction chemotherapy (79% at 49+ months) compared to 5 months in those patients who had ablative therapy +ABMT at relapse after induction treatment. Others have shown that high dose therapy +ABMT can achieve complete remissions and prolonged survival (38% at 2 years, compared to <20% after standard salvage chemotherapy) in relapsed Hodgkin's disease (Jagannath, 1989). Whether it is possible to bridge the gulf between the encouraging results of dose escalation in lymphomas to solid tumours has been the subject of much effort.

In a phase I/II study of 26 patients with advanced breast cancer Jones *et al.* (1987) used within-patient dose escalation of doxorubicin at a starting dose of 25 or 30 mg $m^{-2}$ for 3 days every 4 weeks. They demonstrated an overall response rate of 85% (95% confidence intervals 65–96%) with a 38% CR rate (half of which were pathologically confirmed). Interestingly, although the reported remission rate was high, the median dose of doxorubicin delivered during this study was 99 mg $m^{-2}$ every 4 weeks which in fact is almost identical to the conventional maximum recommended dose of 75 mg $m^{-2}$ 3-weekly (doses which are generally reported to produce remission rates of around 40% (Tormey, 1975)). This difference may result from scheduling differences from other studies or because the maximally tolerated dose for each individual patient was used.

Bronchud *et al.* (1989) reported a dose-escalating study using single agent doxorubicin supported by recombinant granulocyte colony stimulating factor in the treatment of women with advanced breast cancer. They employed a 2-weekly cycle starting at 75 mg $m^{-2}$ escalating up to 150 mg $m^{-2}$. The overall response rate was 80%. All seven patients responded at the two highest dose levels. Non-haematological toxicity was dose limiting.

The use of very high doses of drugs supported by autologous or allogeneic bone marrow transplantation has been shown to produce remissions after failure of standard therapy for solid tumours. Generally, however, such remissions are not prolonged and any effect on survival is uncertain. Antman and Gale (1988) have reviewed the role of high dose chemotherapy supported by autologous bone marrow support in 326 women with breast cancer. Response rates and survival in women with refractory breast cancer were generally low and remissions short-lived. In previously untreated patients response rates were higher (CR rate 56%, OR rate 72%) with longer remissions of 19–55 months. However, the group who responded most favourably are those who showed an initial response to induction therapy followed by high dose chemotherapy and autologous bone marrow rescue with CR and OR rates of 71% and 91% respectively. Treatment mortality was 5–20%.

### Randomised studies of dose and dose intensity

*Dose–response studies*

There are relatively few studies which have set out to prospectively evaluate dose response in a randomised, controlled manner. Most of these studies contain treatment arms which differ in total drug amount administered over the same time. It is therefore difficult to ascertain whether total dose or dose intensity were the important determinants of outcome. Randomised studies of dose are listed in Table I but it is worthwhile considering some of them in more detail.

One of the earliest studies of dose response was made in childhood acute lymphoblastic leukaemia where Pinkel (1971) showed that remission duration was significantly prolonged (15 *vs* 6 months) in children given 'full' rather than 'half' doses of four drug maintainance therapy. However, in a larger study (van Eys, 1989) no difference in outcome was seen for the higher dose arm. Another study (Samson, 1984) revealed that patients with testicular cancer fared better at a higher dose level of cisplatin with an improved CR rate and survival. The dose–response effect of cisplatin in germ cell tumours has recently been confirmed by Ozols (1988), although in this study the waters were muddied by the addition of etoposide in the higher dose arm.

In a study (Cohen, 1977) of 31 patients with previously

**Table I** Prospective randomised studies of dose and dose intensity

| Year | Author | Tumour | No. | Drug[j] | Dose(mg m$^{-2}$ per cycle) | Outcome (low vs high dose) |
|---|---|---|---|---|---|---|
| 1984 | Samson | Testicular | 114 | Cisplatin (vinb & bleo) | 75 vs 120 (4 wkly) | CR 43% vs 63% ($P = 0.03$) survival advantage in high dose arm |
| 1987 | Ozols | Non-semin. germ cell | 52 | Cisplatin[b] vinb (& bleo) | 100, 0.3 vs 200, 0.2 (3 wkly)[b] | CR 67% vs 88% ($P = 0.14$) DFS 33% vs 68% at 4 years ($P = 0.02$) median survival 30 vs >48 months |
| 1971 | Pinkel | ALL in CR | 42 | MP, mtx cyclo & vcr | 350, 20, 200 & 1 vs 175, 10, 100 & 0.5 (wkly) | median remission duration 6 vs 15 months |
| 1989 | van Eys | ALL in CR | 434 | MP and mtx (vcr & pred) | not given[c] | no difference in outcome |
| 1986 | Carmo-Pereira | breast | 48 | Doxorubicin | 35 (8 cycles) vs 70 (16 cycles) (3 wkly) | OR 25% vs 58% (duration 7 vs 14 months) significant survival advantage |
| 1987 | Hortobagyi | breast | 59 | Cyclo, doxo & 5FU | 500, 50 & 1000 vs 1200–1800, 70–100 & 2500 (3 wkly) | no difference in OR, response duration or survival (subsequent analysis revealed similar *delivered* dose intensities in the two arms) |
| 1988 | Tannock | breast | 133 | Cyclo, mtx & 5FU | 300, 20 & 300 vs 600, 40 & 600 mg (3 wkly)[h] | OR 11% vs 30% ($P = 0.03$) median survival 12.8 vs 15.6 months ($P = 0.026$)[i] |
| 1989 | Andrien | breast | 91 | Epirubicin (cyclo & 5FU) | 50 vs 100 (3 wkly) | OR 40% vs 67% ($P < 0.025$) but no survival advantage |
| 1977 | Cohen | SCLC | 32[g] | Cyclo, CCNU & mtx | 1000, 50 & 120 vs 2000, 100 & 180 (6 wks)[d] | OR 45% vs 96% ($P < 0.05$) median survival 5 vs 10.5 months |
| 1985 | Figueredo | SCLC | 103[f] | Doxorubicin cyclo and (vcr) | 50, 1000 vs 60, 1500 (3 wkly)[e] | no difference in CR rate or duration |
| 1986 | Wolff | rec. SCLC | 79 | Etoposide | 300 vs 600 vs 900[a] | only 4 responses altogether, no difference between treatment arms |
| 1986 | Klastersky | NSCLC | 241 | Cisplatin (etoposide) | 60 vs 120 (3–4 wkly) | Or 25% vs 29% ($P$ = n.s.) no survival difference |
| 1981 | Woods | head & neck | 72 | Mtx | 50 vs 500 vs 5000 mg (wkly) | no difference in response or survival |

Doxo, doxorubicin; pred, prednisolone; vcr, vincristine; SCLC, small cell lung cancer; mtx, methotrexate; rec., recurrent; 5FU, 5-fluorouracil; NSCLC, non-small cell lung cancer; cyclo, cyclophosphamide; OR, overall response rate; bleo, bleomycin; CR, complete remission rate; vinb, vinblastine; MP, mercaptopurine.

[a]Retreated on haemotological recovery. [b]High dose arm also received etoposide (500 per cycle). [c]High doses to keep WBC 1,500–3,000, standard doses to keep WBC 3,000–4,500. [d]Doses only escalated between arms for the first six weeks (CCNU given 6 weekly, cyclophosphamide 3 weekly and MTX twice weekly). [e]Escalating to 2,250 in subsequent courses. [f]34 limited and 69 extensive stage disease. [g]27 extensive, 5 local disease. [h]With dose escalation if possible. [i]Survival difference not significant ($P = 0.12$) when adjusted for chance imbalance between two arms for time from first relapse to randomisation. [j]Drugs in brackets at the same doses between treatment arms.

untreated small cell lung cancer randomisation was made between 'high' or low doses of the combination CCNU, cyclophosphamide and methotrexate. Median survival of the high dose group was 10 months compared with 5 months for patients treated with a low dose regimen. The low dose treatment contained 500 mg m$^{-2}$ of cyclophosphamide, a dose which would now be considered to be suboptimal. In a more recent trial (Figueredo, 1985) among 103 patients (randomised to receive 1 or 1.5 g m$^{-2}$ of cyclophosphamide in combination with doxorubicin and vincristine every 3 weeks) no differences were seen in response rate or survival.

Wolff *et al.* (1986) evaluated the dose–response relationships of etoposide in patients with recurrent small cell lung cancer. They used three dose levels of intravenous etoposide 300, 600 and 900 mg m$^{-2}$; 79 patients were treated, divided equally between the three dose levels and despite substantially increased toxicity in the higher dose arm only four partial responses were seen in all patients and they were distributed amongst all dose levels.

In the treatment of advanced non-small cell lung cancer Klastersky *et al.* (1986) conducted a randomised trial comparing a high (120 mg m$^{-2}$) or standard (60 mg m$^{-2}$) dose of cisplatin in combination with etoposide. They reported a 25% objective response rate in the standard arm and 29% in the high dose arm with no improvement in survival.

Hortobagyi (1987) reported the results of a trial comparing conventional and escalating doses of fluorouracil, doxorubicin and cyclophosphamide (FAC) in patients with previously untreated advanced breast cancer. The group of patients receiving escalating dose treatment were further randomised to receive chemotherapy within a protected environment and with the administration of prophylactic antibiotics or in a standard hospital room. Dose escalations were made to 100 mg m$^{-2}$ of doxorubicin and 1800 mg m$^{-2}$ of cyclophosphamide. There were no significant differences in response rates or survival between high and standard dose treatments. Subsequent analysis has, however, revealed that *delivered* dose intensities were similar in the two arms.

### *Dose intensity studies*

One of the few 'positive' studies was that of Carmo-Pereira *et al.* (1986), who performed a randomised study comparing two differing dose intensities of single agent doxorubicin for the treatment of patients with metastatic breast cancer. Forty-eight patients were randomised to receive either 70 mg m$^{-2}$ every 3 weeks for eight courses or 35 mg m$^{-2}$ every 3 weeks for 16 courses. Total dose was therefore the same. There was a response rate of 58% in the more dose intensive arm compared to 25% in the less intensive arm. Response duration and survival were also significantly superior at the higher dose intensity. It may be considered, however, that the less intensive treatment was suboptimal. This highlights an important methodological problem, in that if cytotoxic drug treatment has any effect over no treatment, it will always be possible to choose a small enough dose level to be indistinguishable from no treatment and therefore to be able to demonstrate a dose response effect. Preliminary analysis of a dose intensity study in advanced ovarian cancer where 6 months therapy with a quadruple drug regimen at a high relative dose intensity were compared with 12 months of less intensive treatment (so the total drug doses were identical) has demonstrated a significantly higher overall response rate in the higher dose arm (D. Crowther, personal communication). It is too early, however, to make any comment about response duration or survival.

It can be seen that the few randomised dose studies that are available provide conflicting evidence concerning the correlation between increased dose or dose intensity and an improved response rate and survival.

### Future studies

How may studies be planned in order to address these important and topical questions? Coppin (1987) has elegantly reviewed the problems associated with the description and presentation of drug delivery. Dose and dose intensity although obviously closely linked are variables which need to be addressed separately by prospective randomised studies. Clearly the initial requirement for clinicians attempting to analyse dose intensity within a particular protocol is to record details of actual doses delivered, dose reductions made and treatment delays that occurred. Computer databases need the facility to store this information. Although dose delays to allow haematological recovery are inevitable, the rationale for dose reduction is not established. A recent analysis (N. Thatcher, personal communication) of toxicity occurring within intensive regimens for the treatment of SCLC has established that of 17 infective deaths occurring after a total of 1,966 cycles of chemotherapy, given to 383 patients, only four occurred in those patients previously experiencing life threatening infection and were potentially avoidable by dose reduction. Why, therefore, should doses, be reduced? It would certainly simplify dose–response analysis if no dose reductions were made.

The scheduling of cytotoxic drugs may also differ between regimens where dose and dose intensity are the same and must be considered when two studies are compared. Valdivieso (1984) reported a study of 100 patients with NSCLC. They were randomised to receive doxorubicin at 20 mg m$^{-2}$ weekly or 60 mg m$^{-2}$ 3-weekly with ftorafur, cyclophosphamide and cisplatin (at the same dose and schedule). Response rate, response duration and survival were all greater in the group treated with weekly doxorubicin. Etoposide has also been shown to be highly schedule dependent in SCLC (Slevin *et al.*, 1989).

In summary therefore the hypotheses generated by retrospective analyses regarding dose intensity and total dose have yet to be proven. Further clinical trials are required to elucidate the role of dose intensive regimens in the treatment of malignant disease. As the major dose-limiting toxicity of cytotoxic drug regimens is usually haematological it is likely that the advent of colony stimulating factors will enable us to substantially increase the dose intensity of cytotoxic regimens and provide us with the methods to answer these questions in prospective clinical trials.

### References

ANDRIEN, J.M., CLOSON, M.T., DRIESSCHAERT, P. & 4 others (1989). Dose-Response relationship in advanced breast carcinoma treated with epirubicin ( + Cyclophosphamide and 5 FU). A progress report on a randomized first-line chemotherapy trial. *Proc. ECCO*, **5**, P-1000.

ANTMAN, K. & GALE, R.P. (1988). High dose chemotherapy and autologous bone marrow support for breast cancer. In *Bone Marrow Transplantation: Current Controversies*, Gale, R.P. & Champlin, R. (eds), p 91, Alan R. Liss: New York.

ARMITAGE, J.O. (1989). Bone marrow transplantation in the treatment of patients with lymphoma. *Blood*, **73**, 1749.

BONADONNA, G. & VALAGUSSA, P. (1981). Dose-response effect of adjuvant chemotherapy in breast cancer. *N. Engl. J. Med.*, **316**, 1499.

BONADONNA, G., VALAGUSSA, P. & ROSSI, A. (1985). Ten-year experience with CMF-based adjuvant chemotherapy in resectable breast cancer. *Breast Cancer Res. Treat.*, **5**, 95.

BRONCHUD, M.H., HOWELL, A., CROWTHER, D., HOPWOOD, P., SOUZA, L. & DEXTER, T.M. (1989). The use of granulocyte colony-stimulating factor to increase the intensity of treatment with doxorubicin in patients with advanced breast and ovarian cancer. *Br. J. Cancer*, **60**, 121.

BRUCE, W.R., MEEKER, B.E. & VALERIOTE, F.A. (1966). Comparison of the sensitivity of normal hematopoietic and transplanted lymphoma colony-forming cells to chemotherapeutic agents administered in vivo. *J. Natl Cancer Inst.*, **37**, 233.

BRUFMAN, G., SULKES, A., FUKS, Z. & BIRAN, S. (1983). Cytoxan, methotrexate and 5-fluorouracil (CMF) chemotherapy in metastatic breast cancer: the influence of dose levels and performance status upon response rates and survival. *Proc. ASCO*, **2**, 103.

BULL, J.M., TORMEY, D.C. & LI SHOU, H.M.A. (1978). A randomized comparative trial of Adriamycin versus methotrexate in combination drug therapy. *Cancer*, **41**, 1649.

CARDE, P., MACKINTOSH, F.R. & ROSENBERG, S.A. (1983). A dose and time response analysis of the treatment of Hodgkin's disease with MOPP chemotherapy. *J. Clin. Oncol.*, **1**, 146.

CARMO-PEREIRA, J., COSTA, F.O. & HENRIQUES, E. (1986). Advanced breast carcinoma: A comparison of two dose levels of Adriamycin. *Proc. ASCO*, **5**, 56.

COHEN, M.H., CREAVEN, P.J., FOSSIECK, B.E. & 5 others (1977). Intensive chemotherapy of small cell bronchogenic carcinoma. *Cancer Treat. Rep.*, **61**, 349.

COOPER, R.G. (1969). Combination chemotherapy in hormone resistant breast cancer. *Proc. Am. Assoc. Cancer Res.*, **10**, 15 (abstract).

COOPER, R.G., HOLLAND, J.F. & GLIDEWELL, O. (1979). Adjuvant chemotherapy of breast cancer. *Cancer*, **44**, 793.

COPPIN, C.M.L. (1987). The description of chemotherapy delivery: options and pitfalls. *Semin. Oncol.*, **14**, 34.

FIGUEREDO, A.T., HRYNIUK, W.M., STRAUTMANIS, I., FRANK, G. & RENDELL, S. (1985). Co-trimoxazole prophylaxis during high dose chemotherapy of small-cell lung cancer. *J. Clin. Oncol.*, **3**, 54.

GRISWOLD, D.P., LASTER, W.R., SNOW, M.Y., SCHLABEL, F.M. & SKIPPER, H.E. (1963). Experimental evaluation of potential anticancer agents. *Cancer Res.*, **21** (suppl. 23), 271.

GULATI, S.C., SHANK, B., BLACK, P. & 14 others (1988). Autologous bone marrow transplantation for patients with poor-prognosis lymphoma. *J. Clin. Oncol.*, **6**, 1303.

HENDERSON, I.C., HAYES, D.F. & GELMAN, R. (1988). Dose-response in the treatment of breast cancer: a critical review. *J. Clin. Oncol.*, **6**, 1501.

HORTOBAGYI, G.N., BODEY, G.P., BUZDAR, A.U. & 7 others (1987). Evaluation of high-dose versus standard FAC chemotherapy for advanced breast cancer in protected environment units: a prospective randomized study. *J. Clin. Oncol.*, **5**, 354.

HRYNIUK, W. & BUSH, H. (1984). The importance of dose intensity in chemotherapy of metastatic breast cancer. *J. Clin. Oncol.*, **2**, 1281.

HRYNIUK, W. & LEVINE, M.N. (1986). Analysis of dose intensity for adjuvant chemotherapy trials in stage II breast cancer. *J. Clin. Oncol.*, **4**, 1162.

JAGANNATH, S., ARMITAGE, J.O., DICKE, K.A. & 10 others (1989). Prognostic factors for response and survival after high-dose cyclophosphamide, carmustine, and etoposide with autologous bone marrow transplantation for relapsed Hodgkin's disease. *J. Clin. Oncol.*, **7**, 179.

JONES, R.B., HOLLAND, J.F., BHARDWAJ, S., NORTON, L., WILFINGER, C. & STRASHUN, A. (1987). A phase I-II study of intensive dose adriamycin for advanced breast cancer. *J. Clin. Oncol.*, **5**, 172.

KLASA, R., MURRAY, N. & COLDMAN, A. (1988). Dose intensity meta-analysis of chemotherapy in small cell carcinoma of the lung. *Proc. ASCO*, **7**, 202.

KLASTERSKY, J., SCULIER, J.P., RAVEZ, P. & 10 others (1986). A randomized study comparing a high and a standard dose of cisplatin in combination with etoposide in the treatment of advanced non-small-cell lung carcinoma. *J. Clin. Oncol.*, **4**, 1780.

KWAK, L., OLSHEN, R., HALPERN, J. & HORNING, S.J. (1988). Dose-intensity: relationship to prognostic factors for diffuse large cell lymphoma. *Proc. ASCO*, **7**, 226.

LAGARDE, P., BONICHON, F., AGHBALI, H., DE MASCAREL, I., CHAUVERGNE, J. & HOERNI, B. (1989). Influence of dose intensity and density on therapeutic and toxic effects in Hodgkin's disease. *Br. J. Cancer*, **59**, 645.

LEVIN, L. & HRYNIUK, W.M. (1987). Dose intensity analysis of chemotherapy regimens in ovarian carcinoma. *J. Clin. Oncol.*, **5**, 756.

OZOLS, R.F., IHDE, D.C., LINEHAM, W.M., JACOB, J., OSTCHEGA, Y. & YOUNG, R.C. (1988). A randomized trial of standard chemotherapy *v* a high dose chemotherapy regimen in the treatment of poor prognosis nonseminomatous germ-cell tumors. *J. Clin. Oncol.*, **6**, 1031.

PALMER, M., BELCH, A., HANSON, J. & BROX, L. (1988). Dose intensity analysis of melphalan and prednisolone in multiple myeloma. *J. Natl Cancer Inst.*, **80**, 414.

PINKEL, D., HERNANDEZ, K., BORELLA, L. & 4 others (1971). Drug dosage and remission duration in childhood lymphocytic leukaemia. *Cancer*, **27**, 247.

REDMAN, C., FISHER, B. & WIEAND. S. (1983). The methodological dilemma in retrospectively correlating the amount of chemotherapy received in adjuvant therapy protocols with disease-free survival. *Cancer Treat. Rep.*, **67**, 519.

SAMSON, M.K., RIVKIN, S.E., JONES, S.E. & 5 others (1984). Dose-response and dose-survival advantage for high versus low-dose cisplatin combined with vinblastine and bleomycin in disseminated testicular cancer. *Cancer*, **53**, 1029.

SCHABEL, F.M., GRISWOLD, D.P., CORBETT, T.H. & LASTER, W.R. (1984). Increasing the therapeutic response rates to anticancer drugs by applying the basic principles of pharmacology. *Cancer*, **54**, 1160.

SLEVIN, M.L., CLARKE, P.I., JOEL, S.P. & 6 others (1989). A randomized trial to evaluate the effect of schedule on the activity of etoposide in small-cell lung cancer. *J. Clin. Oncol.*, **7**, 1333.

TANNOCK, I.F., BOYD, N.F., DEBOER, G. & 6 others (1988). A randomized trial of two dose levels of cyclophosphamide, methotrexate, and fluorouracil chemotherapy for patients with metastatic breast cancer. *J. Clin. Oncol.*, **6**, 1377.

TORMEY, D.C. (1975). Adriamycin (NSC-123127) in breast cancer: an overview of studies. *Cancer Chemother. Rep.*, **6**, 319.

VALDIVIESO, M., BURGESS, M.A., EWER, M.S. & 6 others (1984). Increased therapeutic index of weekly doxorubicin in the therapy of non-small cell lung cancer: a prospective, randomized study. *J. Clin. Oncol.*, **2**, 207.

VAN EYS, J., BERRY, D., CRIST, W. & 4 others (1989). Treatment intensity and outcome for children with acute lymphocytic leukaemia of standard risk. *Cancer*, **63**, 1466.

WOLFF, S.N., BIRCH, R., SARMA, P. & GRECO, F.A. (1986). Randomized dose-response evaluation of etoposide in small cell carcinoma of the lung: a Southeastern Cancer Study Group trial. *Cancer Treat. Rep.*, **70**, 583.

WOODS, R.L., FOX, R.M. & TATTERSALL, M.H.N. (1981). Methotrexate treatment of squamous-cell head and neck cancers: dose-response evaluation. *Br. Med. J.*, **282**, 600.